# Effect of freezing treatment of soybean on soymilk nutritional components, protein digestibility, and functional components

**DOI:** 10.1002/fsn3.2524

**Published:** 2021-09-09

**Authors:** Meng‐jia Li, Ru‐ge Cao, Li‐tao Tong, Bei Fan, Ruo‐qi Sun, Li‐ya Liu, Feng‐zhong Wang, Li‐li Wang

**Affiliations:** ^1^ Institute of Food Science and Technology Chinese Academy of Agricultural Sciences Key Laboratory of Agro‐Products Processing Ministry of Agriculture and Rural Affairs Beijing China; ^2^ School of Food Science and Engineering Tianjin University of Science & Technology Tianjin China

**Keywords:** chymotrypsin inhibitor activity, nutritional components, protein digestibility, soymilk, trypsin inhibitor activity

## Abstract

Soymilk is a popular beverage in many countries owing to its nutrition and health effects. To increase household consumption of soymilk, instant soybeans were developed by freezing and subsequent drying pretreatment, which overcome the time‐consuming need of soaking during soymilk preparation for home making. However, compared with the traditional soymilk making, the nutritional quality and functional properties of this soymilk made from the soybean by direct grinding in water without soaking are not clear yet. Soymilk made from untreated soybeans, soaked soybeans, and soaking, freezing, and air‐drying soybeans (FADTS) were compared on their properties including nutritional components, in vitro protein digestibility, and functional components. It was found that FADTS was the best at extracting lipid and Ca, good at extracting of protein, carbohydrate, oligosaccharides, Fe, phytic acids, and tannins, and in producing soymilks with highest in vitro protein digestibility. The soluble protein and protein digestibility of FADTS (4 day) increased significantly from 44.4% and 78.5% of control to 56.2% and 85.0%, respectively. Soymilk from 4 days FADTS contained similar protein content and higher Fe content (4.40 mg/kg) compared to soaked sample (3.82 mg/kg). The results revealed that FADTS performed better at producing soymilk than untreated and soaked soybeans.

## Graphical abstract

Soymilk made from untreated soybeans, soaked soybeans, and soaking, freezing, and air‐drying soybeans (FADTS) were compared on their properties including nutritional components, in vitro protein digestibility, and functional components. The results revealed that FADTS performed better at producing soymilk than untreated and soaked soybeans. FADTS can be served as an instant soybean product for producing nutritional soymilk without time‐consuming soaking, which facilitate the life of modern people.

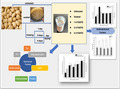

## 1 INTRODUCTION

Soymilk is a popular beverage in many countries, especially in Asian countries (Ono, 2008). It is rich in proteins, iron, unsaturated fatty acids, and niacin, but low in fat, carbohydrates, and calcium (Zhao et al., 2018). The soymilk benefits people who are lactose‐intolerant or live in the regions where the supply of animal milk is inadequate. In addition, soymilk also contains some functional components such as Kunitz trypsin inhibitor (KTI), Bowman–Birk inhibitor (BBI), phytic acid, and tannins. Kunitz and Bowman–Birk are the most studied inhibitor families and are found in abundance in various leguminous plants (Oliveira et al., 2012). On one hand, they are considered as antinutritional components, which may negatively affect the nutritional quality and digestibility of soybean protein (Nagao et al., 2017). On the other hand, KTI and BBI are reported as potential chemopreventive agents; the important physiological roles and potential beneficial functions are also increasingly being recognized (Kennedy, 1998; Kobayashi et al., 2004; Zhou et al., 2017). It has been proved that protease inhibitors can regulate endogenous enzymes and against proteolytic action of the digestive enzymes of pathogens and pests during seed development. Phytic acid, by virtue of forming a unique iron chelate, may lower the incidence of colonic cancer and protect against other inflammatory bowel diseases (Chen et al., 2018). And tannins, which are found in many plant‐based foods and beverages, are potentially very important biological antioxidants (Tanaka et al., 2018). Although these antinutritional factors are also functional components, their impact on human body is controversial. However, from the perspective of improving the nutritional value of food, it is very necessary to properly remove these antinutritional factors in the processing of soybean products.

Traditionally, soymilk is supplied as commercial products. However, it has become a regular home‐made food along with the invention of soymilk grinder. Untreated soybeans are commonly used to make soymilk using soymilk blender by consumers (without time‐consuming soaking). But it is found that soymilk from untreated soybeans has lower solid content and worse stability than soymilk from soaked soybeans (Wang et al., 2013). To increase household consumption of soymilk, some instant soybeans were developed by different pretreatment which overcome the time‐consuming need of soaking during soymilk preparation for home making. Freezing pretreatment as an effective technique has been used to alter the mechanical properties of the cell material because tissue damage could modify the texture of food and improve processing properties. It was reported that freezing could significantly improve the water absorption rate in rehydration and decrease cooking hardness of black beans (Gao et al., 2011). Similarly, the freezing technology can also be used to produce instant soybeans for soymilk grinder. In our previous study, we developed a kind of instant soybean product (termed as FADTS in this study) by a combined treatment of soaking, freezing, and air‐drying, and its soymilk had higher solid content and better stability than soymilk from untreated soybeans (Wang et al., 2013). Inactivation of trypsin inhibitors, along with deleterious enzymes, antinutrition factor and increasing the protein quality by improving its functionality and digestibility are the most important factors to be considered in crucial stage in manufacturing of soy products. However, the properties of soymilk made from freezing soybeans have not been studied yet. In this work, soymilks prepared from FADTS for different freezing time (1 day, 2 days, and 4 days) was compared to soymilk made from untreated and soaked soybeans. The properties including protein, lipid, carbohydrate, amino acids, oligosaccharides, Ca, Fe, in vitro protein digestibility, and some functional components (KTI, BBI, phytic acid, and tannins) were evaluated among different methods.

## 2 MATERIALS AND METHODS

### 2.1 Preparation of soaking, freezing, and air‐drying treated soybeans (FADTS)

Soybeans (Zhonghuang No. 13), harvested in 2011, were obtained from Chinese Academy of Agricultural Sciences, which contained about 39 g of protein and 17 g of lipid in 100 g soybeans (moisture content, 14%). They were stored at 4°C until use. One hundred gram of soybeans were washed and soaked in the de‐ionized (DI) water at 20°C for 10 h. The soaked soybeans were placed in a plastic draining basket to remove excess water and frozen at −5°C for 1, 2, and 4 days (BCD‐278AZ, Hefei Meiling Co., Ltd, China). The soybeans were dried at 45°C in an air‐dryer (DGX‐9073 B‐1, Shanghai FuMa Text Equipment Co., Ltd, China). Weigh once every hour until the sample mass drops to 100 ± 0.01g and stop drying. Then, they were stored at 4°C until use.

### 2.2 Soymilk preparation

Untreated soybeans (100 g), FADTS (100 g) or soaked soybeans (100 g soybean soaked at 20°C water 300 ml for 10 h) were added into a soymilk grinder (Model FSM‐100, Shenyang Machinery No. 3 Factory, China), and then, DI water was added to make the total weight of 1,000 g. The mixture was ground for 5 min and then filtered through a 100‐mesh sieve. The filtrate was termed raw soymilk, which was further heated at >95°C for 10 min by a thermostatic water bath (Beijing Changan Equipment Co., Ltd, China).

### 2.3 Determination of protein, lipid, carbohydrate, Ca, and Fe contents

Protein content was determined following the Bradford method (Bradford, 1976). Lipid was determined with AACC method 30–25 (AACC, 2000). The freeze‐drying soymilk was extracted for 6 h with petroleum ether with an Automated Soxhlet Extractor (SZF‐06C, Zhejiang TuoPu Equipment Co., Ltd, China). The determination of carbohydrate was done according to the method described by Laurentin and Edwards (2003). Ca and Fe were determined by atomic absorption spectroscopy (Perkin Elmer, Model AA800, USA) (Ferreira & Tarley, 2021).

### 2.4 Amino acid analysis

The freeze‐dried soymilk samples (200 ml) were defatted and then hydrolyzed in 6 *M* (mol/L) HCl (110°C, 24 h), or hydrolyzed in 6 N NaOH (110°C, 24 h) for tryptophan. Amino acid composition was determined using an automatic amino acid analyzer (L‐8500; Hitachi Ltd., Tokyo, Japan). The amino acids were separated on Hitachi High‐Tech 2622PH Column (4.6 × 60 mm) using sodium citric acid buffer at pH 2.2, a flow rate of 0.225 ml/min, a column temperature of 57°C according to the procedure described by Yang and Zhang (2009). Individual amino acids were quantified on the basis of amino acid standard (AAS18, Sigma Chemical Co., USA).

### 2.5 Soluble protein

The soluble protein was obtained by the method described by Ono et al. (1991), with some modifications. Briefly, 6 ml of soymilk was added into centrifuge tube with pipette, and then, 3 ml of 50% (w/w) sucrose solution was carefully injected to the tube bottom. After being treated by ultracentrifugation (156,000 × *g*, 30 min), soymilk was separated into five parts: floating (oil bodies), supernatant (soluble protein), protein particle layer, sucrose solution, and little precipitate on the tube bottom. Each 2 ml of supernatant, containing <40 nm soluble protein, was carefully collected and protein concentration was determined. The percentage of soluble protein in whole soymilk protein was calculated.

### 2.6 Oligosaccharides analysis

Oligosaccharide contents were measured according to the method by Hou et al. (2000) and using a high‐performance liquid chromatography (HPLC) system (LC‐20 AB, Shimadzu, Japan) equipped with a Waters carbohydrate high‐performance column (4.6 × 250 mm). The oligosaccharides were eluted with water/acetonitrile (1/4, v/v) for 20 min, at 40°C, with a flow rate of 0.8 ml/min.

Eluted oligosaccharides in the effluent were quantified with a refractive index (RI) detector using sucrose, stachyose, and raffinose as standards (Sigma, Chemical Co., USA).

### 2.7 Trypsin inhibitor activity (TIA) and chymotrypsin inhibitor activity (CIA) assays

Trypsin inhibitor activity and CIA of soymilks were determined according to the methods by Xu et al. (2012).

### 2.8 Phytic acid

Phytic acid was determined following the method described by Latta and Eskin (1980) with slight modifications. Phytic acid was first concentrated on anion exchanged resin. Then, inorganic phosphate was eluted with 0.05 mol/L NaCl solution and the phytate was eluted with 0.7 mol/L NaCl solution.

Phytate content in elute was then determined by reaction with a solution containing 0.03% FeCl_3_•6H_2_O and 0.3% sulfosalicylic acid in distilled water. Final color development was measured at 500 nm with a spectrophotometer (UV/V 2,450, Shimadzu Company, Japan).

### 2.9 Tannins

Tannin was assayed according to the modified vanillin‐HCl method (Price et al., 1978). Catechin (Sigma Chemical Co., USA) was used as standard, and tannin concentration was expressed in mg of catechin equivalent.

### 2.10 Determination of in vitro protein digestibility

Digestibility of soymilk was assessed using two‐step digestion methods as described by Iwami et al. (1986), with some modifications. Pepsin (Sigma, P7000, 1:10,000, 600–1,000 units/mg) and trypsin (Genview, DH355‐1, 1:250) were used for in vitro digestion study. Briefly, 5 ml of soymilk was adjusted to pH 2.0 with HCl (1.0 mol/L), and then, pepsin (enzyme‐to‐substrate ratio of 1:100) was added and incubated in a water bath at 37°C. After 3 h, pH was adjusted to 7.0 with 1.0 mol/L NaOH to stop the enzymatic reaction. The second digestion was carried out with trypsin (enzyme‐to‐substrate ratio of 1:200) at 37°C for 3 h. Subsequently, the digested mixtures were mixed with 10 ml of 10% (w/v) trichloroacetic acid (TCA) and centrifuged (6,000 g, 30 min) to obtain the precipitate which was then dispersed into 10 ml of TCA (10%, w/v) and centrifuged again. The nitrogen content was determined by Kjeldahl method, and protein digestibility of soymilk was calculated according to the following equation (Tang, 2007):

(1) $$In\;vitro\;\text{protein digestibility}\;=\;\left(N_{0}‐N_{t}\right)\times 100/N_{\mathrm{total}}$$

where *N_0_* (mg) is the TCA‐insoluble nitrogen in the sample, *N_t_* (mg) is the TCA‐insoluble N nitrogen after digestion and *N_total_* (mg) is the total nitrogen of sample.

### 2.11 Statistical analysis

Data were analyzed by analysis of variance (ANOVA) using SPSS version 17.0 (SPSS, Inc., Chicago, USA). Comparisons of means were made by Duncan test. *p* ≤ .05 or less was considered significant. Data were expressed as numerical means and standard deviations (mean ± *SD*). Each sample was analyzed in triplicate.

## 3 RESULTS AND DISCUSSION

### 3.1 Lipid, protein, and carbohydrate of soymilks

The lipid, protein, and carbohydrate of soymilks from untreated soybeans, soaked soybeans, and FADTS (frozen for 1, 2, and 4 days) were determined. Table 1 shows that soymilk from untreated soybeans contains the lowest lipid (10.19 mg/ml), protein (18.64 mg/ml), and carbohydrate (16.55 mg/ml); soymilk from soaked soybeans contains the highest carbohydrate (21.58 mg/ml) and protein (24.64 mg/ml). The lipid, protein, and carbohydrate of soymilk from FADTS are tended to increase along with frozen days. Soymilk from 1 day FADTS contains more lipid than the soymilk from soaked soybeans, while soymilk from 4 days FADTS contains slightly lower protein and carbohydrate than the soymilk from soaked soybeans. Totally, it was suggested that 4 days FATDS was better than untreated and soaked soybeans at the extraction efficiencies of lipid and protein, which should be resulted from the destructive effect of freezing treatment on the soaked soybean microstructure. It is known that slow freezing could result in extensive cell rupture owing to the ice crystal growth. Previous research also showed that when soybeans were frozen, compared with untreated samples, well‐shaped protein storage vacuoles were damaged significantly and some oil bodies, an organelle where lipid is mainly stored in, were coalesced into larger ones (Wang et al., 2013). The results above showed that more proteins could be extracted from FADTS. It was considered that more soybean storage proteins were released into soymilk the cell structure loose induced by the freezing, and air‐drying.

**TABLE 1:** Protein, lipid, and carbohydrate contents in soymilk from different treatments

Conditions	Protein (mg/ml)	Lipid (mg/ml)	Carbohydrate (mg/ml)
Untreated	18.64 ± 0.03^a^	10.19 ± 0.01^a^	16.55 ± 0.02^a^
Soaked	24.64 ± 0.54^d^	13.90 ± 0.03^b^	21.58 ± 0.54^d^
1 day FADTS	20.38 ± 0.81^b^	14.52 ± 0.38^b^	18.10 ± 0.11^b^
2 days FADTS	22.31 ± 0.48^c^	15.21 ± 0.58^c^	19.65 ± 0.22^c^
4 days FADTS	24.16 ± 0.54^d^	15.76 ± 0.17^c^	19.24 ± 0.32^c^

Note

Values are means ± standard deviations (*n* = 3). Means with different letters in the same column are significantly different at *p* < .05.

### 3.2 Soluble protein of soymilks

**FIGURE 1:**
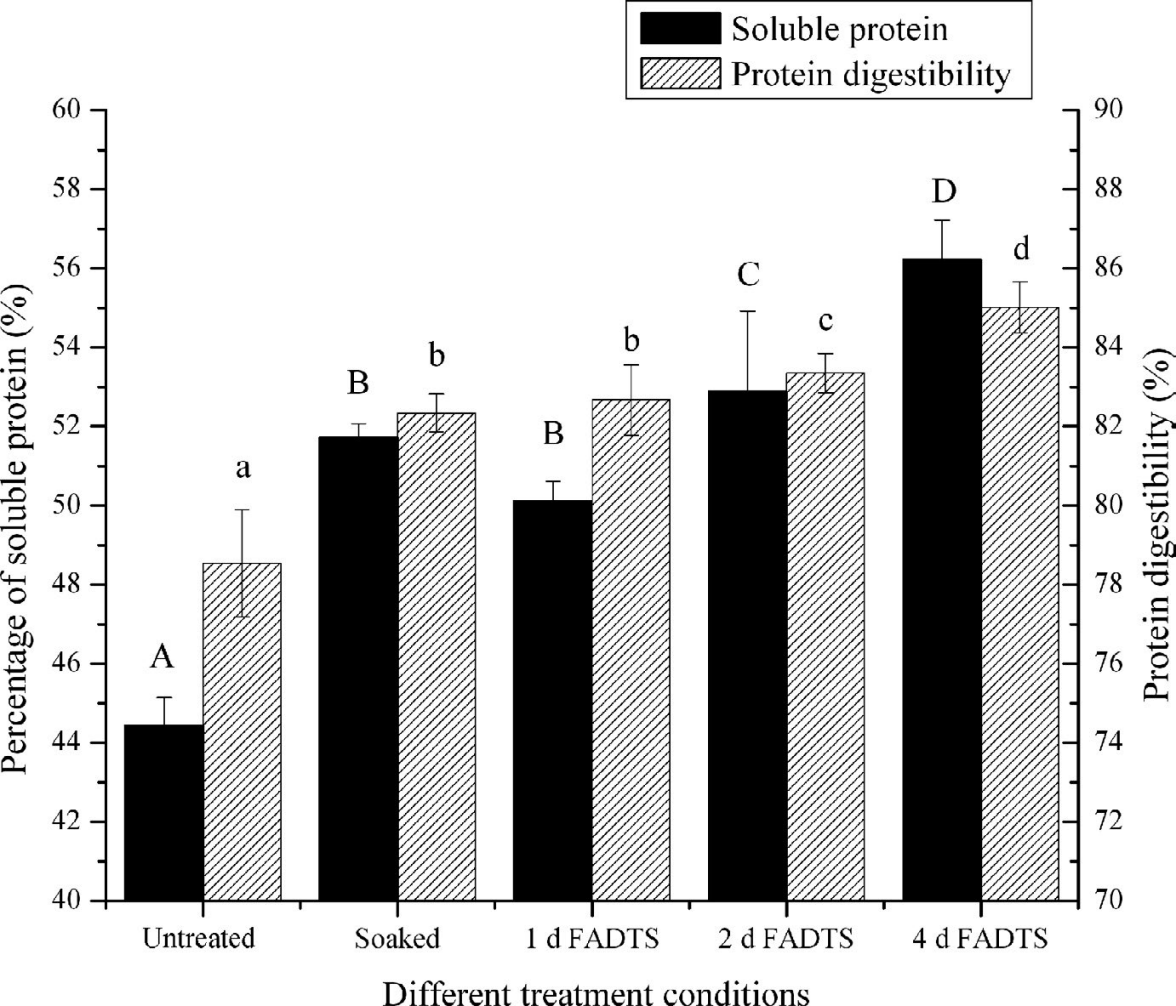
Effect of freezing on the percentages of soluble protein in whole protein and in vitro protein digestibility of soymilk

It was reported (Ono et al., 1991) that soymilk protein could be divided into small soluble protein (<40 nm) and large protein particles (>40 nm, average value about 100 nm) by ultracentrifugation (156,000 g, 30 min). Figure 1 shows that the percentages of soluble protein in soymilk protein are about 44.4%, 51.7%, and 50.1% for untreated soybeans, soaked soybeans, and 1 day FADTS, respectively. The percentage of soluble protein increased from about 50.1% to 56.2% when frozen times of FADTS increased from 1 day to 4 days. It was considered that soymilk protein from FADTS might be easily digested owing to its higher percentage of small soluble protein, which had a larger surface‐to‐volume ratio.

### 3.3 Amino acid compositions of soymilks

Soy proteins have been widely used to formulate foods with a goal of improving their nutritional and functional qualities due to the high protein level and well‐balanced amino acid composition. Table 2 shows the amino acid compositions of soymilks from different treatments. It was clear that glutamic acid/glutamine, aspartic acid/asparagine, histidine, and leucine were the major contributors to amino acids. The total amino acid content in soymilk from soaked soybeans was 16.13 mg/ml, significantly higher than that of untreated soybeans (14.21 mg/ml), and FADTS gave intermediate values (14.69–15.40 mg/ml), showing a similar trend as the soymilk protein. The results showed that soaked soybeans were the best for the amino acids of soymilk, followed by 4 days FADTS, 2 days FADTS, 1 day FADTS and untreated soybeans. FADTS showed a disadvantageous result at the soymilk tryptophan, which might be induced by the combined treatment of soaking, freezing and air‐drying. This was because some treatments (i.e., soaking, boiling, and cooking) could decrease the concentrations of isoleucine‐, tryptophan‐, and sulfur‐containing amino acids when mung bean was similarly treated (Mubarak, 2005).

**TABLE 2:** Changes of amino acid contents in soymilk from different treatments

Amino acids (mg/g protein)	Conditions				
	Untreated	Soaked	1 day FADTS	2 days FADTS	4 days FADTS
Aspartic acid	1.50 ± 0.043^a^	1.69 ± 0.045^e^	1.52 ± 0.048^b^	1.56 ± 0.058^c^	1.60 ± 0.009^d^
Threonine	0.47 ± 0.011^a^	0.53 ± 0.077^c^	0.49 ± 0.015^b^	0.50 ± 0.017^b^	0.51 ± 0.034^b^
Serine	0.62 ± 0.016^a^	0.70 ± 0.010^d^	0.65 ± 0.019^b^	0.66 ± 0.013^b^	0.67 ± 0.012^c^
Glutamic acid	2.64 ± 0.032^a^	3.01 ± 0.058^e^	2.77 ± 0.015^b^	2.81 ± 0.023^c^	2.87 ± 0.020^d^
Glycine	0.51 ± 0.013^a^	0.58 ± 0.038^d^	0.54 ± 0.022^b^	0.55 ± 0.020^b^	0.56 ± 0.028^c^
Alanine	0.53 ± 0.021^a^	0.60 ± 0.034^d^	0.55 ± 0.018^b^	0.56 ± 0.019^b^	0.57 ± 0.047^c^
Cystine	0.27 ± 0.009^a^	0.30 ± 0.018^c^	0.27 ± 0.011^a^	0.28 ± 0.012^b^	0.30 ± 0.026^c^
Valine	0.57 ± 0.017^a^	0.64 ± 0.043^e^	0.59 ± 0.030^b^	0.61 ± 0.037^c^	0.63 ± 0.046^d^
Methionine	0.10 ± 0.069^a^	0.13 ± 0.031^c^	0.12 ± 0.011^b^	0.12 ± 0.017^b^	0.14 ± 0.054^d^
Isoleucine	0.58 ± 0.010^a^	0.66 ± 0.037^e^	0.60 ± 0.014^b^	0.62 ± 0.011^c^	0.64 ± 0.006^d^
Leucine	1.00 ± 0.008^a^	1.13 ± 0.049^d^	1.04 ± 0.013^a^	1.07 ± 0.002^b^	1.10 ± 0.006^c^
Tyrosine	0.48 ± 0.016^a^	0.54 ± 0.035^d^	0.51 ± 0.008^b^	0.51 ± 0.018^b^	0.52 ± 0.062^c^
Phenylalanine	0.70 ± 0.010^a^	0.78 ± 0.019^e^	0.72 ± 0.009^b^	0.74 ± 0.022^c^	0.76 ± 0.014^d^
Lysine	0.86 ± 0.009^a^	0.91 ± 0.035^b^	0.91 ± 0.074^b^	0.85 ± 0.014^a^	0.87 ± 0.063^a^
Histidine	1.98 ± 0.025^a^	2.23 ± 0.008^d^	2.06 ± 0.026^b^	2.08 ± 0.045^b^	2.14 ± 0.017^c^
Tryptophan	0.48 ± 0.013^c^	0.53 ± 0.021^d^	0.41 ± 0.033^a^	0.46 ± 0.011^b^	0.46 ± 0.022^b^
Arginine	0.34 ± 0.044^a^	0.38 ± 0.016^c^	0.35 ± 0.021^a^	0.36 ± 0.015^b^	0.37 ± 0.041^b^
Proline	0.64 ± 0.017^a^	0.73 ± 0.014^e^	0.67 ± 0.021^b^	0.69 ± 0.027^c^	0.70 ± 0.008^d^
Total	14.21 ± 0.009^a^	16.13 ± 0.032^e^	14.69 ± 0.021^b^	15.01 ± 0.017^c^	15.40 ± 0.041^d^

Note

Measurements were performed in triplicate. Values are means ± standard deviations (*n* = 3). Means with different letters in the same column are significantly different at *p* < .05.

### 3.4 Oligosaccharides (sucrose, stachyose, and raffinose) of soymilks

Soybean oligosaccharides have prebiotic effects and are related to several health benefits, such as lowing of blood cholesterol level, enhancement of minerals absorption, and prevention of some types of cancer (Mussatto & Mancilha, 2007; Roberfroid, 2007). However, high levels of nondigestible oligosaccharides (stachyose & raffinose) can cause flatulence and abdominal pain (Girigowda & Mulimani, 2006), since they tend to be degraded into carbon dioxide, hydrogen, and methane by bacteria in the human intestine. Table 3 shows that soymilk from soaked soybeans has the highest oligosaccharides contents while FADTS is the lower at the extraction of oligosaccharides compared to the soaked soybeans, but higher than untreated soybeans, which is consistent with the carbohydrate in Table 1. The oligosaccharide contents of soymilk from FADTS showed a gradual increase trend with frozen days. These results showed that the oligosaccharides of soymilk from FADTS had an intermediate value between soaked and untreated soybeans.

**TABLE 3:** Oligosaccharide (sucrose, raffinose, stachyose) contents in soymilk from different treatments

Conditions	Concentrations (mg/ml)		
	Sucrose	Raffinose	Stachyose
Untreated	3.67 ± 0.01^a^	0.50 ± 0.01^a^	5.15 ± 0.27^a^
Soaked	6.25 ± 0.09^d^	0.75 ± 0.03^d^	7.35 ± 0.71^e^
1 day FADTS	4.66 ± 0.01^b^	0.50 ± 0.05^a^	5.43 ± 0.07^b^
2 days FADTS	4.92 ± 0.05^b^	0.51 ± 0.01^b^	5.66 ± 0.44^c^
4 days FADTS	5.06 ± 0.08^c^	0.56 ± 0.06^c^	6.17 ± 0.15^d^

Note

Values are means ± standard deviations (*n* = 3). Means with different letters in the same column are significantly different at *p* < .05.

### 3.5 Ca and Fe of soymilks

Table 4 showed that soymilk from FADTS contains the highest Ca contents (63.10–66.92 mg/kg), followed by soaked (61.21 mg/kg) and untreated soybeans (53.80 mg/kg), revealing the same trend as soymilk lipid. It was reported that soymilk lipid existed as oil bodies (Chen et al., 2009), which contained Ca of about 32.68–43.94 mg/100 g of oil bodies (dry basis) (Zhao et al., 2013). This might be used to explain why soymilk Ca showed the same trend as lipid. Soymilk from untreated soybeans contained the lowest Fe (3.45 mg/kg), while soymilk from soaked soybeans contained Fe of 3.82 mg/kg, which was higher than the 1 day and 2 days FADTS but lower than 4 days FADTS. It clearly revealed that 4 days FADTS was the best at the Ca and Fe extraction efficiency.

**TABLE 4:** Ca and Fe contents in soymilk from different treatments

Conditions	Contents (mg/kg)	
	Ca	Fe
Untreated	53.80 ± 0.05^a^	3.45 ± 0.45^a^
Soaked	61.21 ± 0.17^b^	3.82 ± 0.19^c^
1 day FADTS	63.10 ± 0.00^c^	3.63 ± 0.19^b^
2 days FADTS	68.84 ± 0.32^e^	3.81 ± 0.02^c^
4 days FADTS	66.92 ± 0.11^d^	4.40 ± 0.22^d^

Note

Values are means ± standard deviations (*n* = 3). Means with different letters in the same column are significantly different at *p* < .05.

### 3.6 Trypsin inhibitor activity and chymotrypsin inhibitor activity

Soybean trypsin inhibitors mainly include Kunitz trypsin inhibitor (KTI) and Bowman–Birk inhibitor (BBI). The former one has the trypsin inhibitor activity (TIA), while the later one possesses chymotrypsin inhibitor activity (CIA) as well as TIA. Trypsin and chymotrypsin are important proteases in animal digestive tract and enable people to digest protein into dipeptides and tripeptides (Guerrero‐Beltrán et al., 2009). Therefore, maximum inactivation of KTI and BBI is considered necessary in the processing of soybean products. However, some researchers reported that KTI and BBI were potential chemopreventive agents (Kennedy, 1998; Kobayashi et al., 2004). And Amigo‐Benavent et al. (2013) tried to make functional orange juice by adding BBI as a natural functional food ingredient. Therefore, it was considered that KTI and BBI might exert some positive points to the soymilks. TIA and CIA values of untreated, soaking, and freezing pretreated group are depicted in Table 5, which shows that soymilk from untreated soybeans has the highest TIA (921.25 ± 21.04), followed by FADTS (787.63 ± 48.58–859.38 ± 42.07) and soaked soybeans (726.00 ± 42.11). And it is found that the TIA of soymilk from FADTS is increased with the prolonging of frozen days. The CIA of soymilk reduced by 48.53 unit/ml after 1‐day freezing treatment when compared to the control groups, which is also the lowest CIA in the treated group. Similarly, with the increase of freezing days, CIA increased gradually. As the processing time was further increased to 4 days, the CIA present in soymilk rose to 452.75 unit/ml, which is an increase of 3.24% compared to the soaked group. The increase in TIA and CIA can be explained by the tissue structure of soybean is fully destroyed with the extension of freezing time, and more protein is released into soymilk during grinding (Table 1). However, the protein aggregation and denaturation caused by freezing treatment resulted in a lower TIA in FADTS than in the untreated group. The secondary, tertiary, and quaternary structural changes of protein are easy to change under the extreme environmental conditions of freezing, which is manifested in the aggregation or degradation of protein bodies, which is reflected in the research of Wang et al. (2013). After freezing treatment, most protein bodies combine with each other to form larger irregular protein aggregates, and with the extension of freezing time, the originally regular protein bodies disappear. KTI and BBI are essentially proteins, which may also participate in the formation of protein aggregates (including large and small aggregates), thus affecting their activity and inactivating them (Xu et al., 2012). In addition, the processing methods involving hot air‐drying treatment have become the secondary reason to diminish TIA. It has been suggested that heat can alter trypsin inhibitor's molecular structure, making their disulfide bonds more prone to undergo reduction reactions (Avilés‐Gaxiola et al., 2018). The least that can be concluded from this research is that FADTS was better at producing soymilk than untreated soybeans, worse than soaked soybeans in the case of TIA inactivation, but for CIA inactivation, the effect of FADTS is better than soaking group if the appropriate freezing treatment time is selected.

**TABLE 5:** Trypsin inhibitor activity, CIA, phytic acid, and tannins contents in soymilk from different treatments

Conditions	TIA (unit/ml)	CIA (unit/ml)	Phytic acid (mg/ml)	Tannins (mg/ml)
Untreated	921.25 ± 21.04^e^	448.88 ± 15.91^d^	0.41 ± 0.00^a^	0.10 ± 0.01^a^
Soaked	726.00 ± 42.11^a^	438.53 ± 11.31^c^	0.99 ± 0.01^d^	0.13 ± 0.00^d^
1 day FADTS	787.63 ± 48.58^b^	400.35 ± 9.90^a^	0.59 ± 0.01^b^	0.11 ± 0.00^b^
2 days FADTS	827.75 ± 46.78^c^	411.08 ± 1.41^b^	0.60 ± 0.00^b^	0.11 ± 0.00^b^
4 days FADTS	859.38 ± 42.07^d^	452.75 ± 10.00^d^	0.61 ± 0.03^c^	0.12 ± 0.01^c^

Note

Values are means ±standard deviations (*n* = 3). Means with different letters in the same column are significantly different at *p* < .05.

### 3.7 Phytic acid and tannins of soymilks

Phytic acid, on one hand, can interact with proteins, peptides, or cations such as calcium, magnesium, copper, zinc, and iron, which inferiors the bioavailability of minerals. On the other hand, it can also bind endogenous enzymes such as chymotrypsin and trypsin in the gastrointestinal tract (Singh & Krikorian, 1982), thus inhibiting the effects of proteolytic enzymes and decreasing the digestibility of protein. As shown in Table 5, soymilks from soaked and untreated soybeans contain the highest (0.99 mg/ml) and lowest (0.41 mg/ml) phytic acid contents, respectively. Meanwhile, the phytic acid contents ranged from 0.59 to 0.61 mg/ml as the frozen days increased from 1 to 3 days, lower than that of soymilk from soaked soybeans and higher than that of soymilk from untreated soybeans, showing the same trend as protein and carbohydrate. Generally, under the same grinding condition, soaked samples gained more cell material due to its soft texture (Shimelis & Rakshit, 2007). For FADTS, during grinding, the disrupted soybean structure caused by the freezing treatment made more phytic acid release into the soymilk than untreated soybeans, but less than soaked soybeans.

Tannins, a kind of plant polyphenols, have received considerable attention because of their ability to interact with protein to decrease protein digestibility. Table 5 reveals that the tannins of different soymilks show the same trend as phytic acid. Soymilk from soaked soybeans contained the highest tannins (0.13 mg/ml), followed by FADTS (0.11–0.12 mg/ml) and untreated soybeans (0.10 mg/ml), and the small differences among them should be resulted from the low content of tannins in soybeans (Egounlety & Aworh, 2003). Similar to KTI and BBI, tannins and phytic acid were also considered as functional components by some researchers (Chen et al., 2018; Tanaka et al., 2018), so they might also exert some positive points to the soymilks.

### 3.8 In vitro protein digestibility of soymilks

In addition to analyzing the composition of amino acids, the nutritional value of soybean products also depends on the digestibility of protein. And the digestibility of soymilk protein is usually evaluated by measuring the in vitro protein digestibility. The in vitro protein digestibility of soymilks is shown in Figure 1. Soymilks from FADTS (1, 2, and 4 days) exhibited the in vitro protein digestibility of about 82.6, 83.0, and 85.0%, respectively, while soymilks from untreated and soaked soybeans were about 78.5% and 82.3%, respectively. The results revealed that FATDS was the best at improving in vitro protein digestibility, which was enhanced by the prolonging of frozen days. It was considered that the high protein digestibility should be derived from the combined effects of the increasing percentage of soluble protein (small size), relatively low TIA and CIA as well as the relatively low contents of phytic acid and tannins of soymilks from FADTS. On the one hand, it can be seen from Figure 1 that the content of soluble protein in FADTS is higher than that in the other two groups, while many studies believe that proteins with small particle size are easier to be digested by humans and animals and increase digestibility (Mavromichalis et al., 2000). In addition, the contents of TIA, CIA, phytic acid, and tannin in soymilk decreased after freezing treatment, and the passivation of these antinutritional factors is directly proportional to the improvement of protein digestibility, so it can be considered that freezing treatment can improve the nutritional value of soymilk (Skv et al., 2020).

## 4 CONCLUSION

In this study, soymilk was made by three different methods: 1) the traditional method by using soaked soybeans (soaked soybeans); 2) a well‐used method for soymilk grinder by using untreated soybeans; 3) a new method by using FADTS. It was found that soaked soybeans were the best at extracting protein and carbohydrate, oligosaccharides, phytic acids, and tannins; untreated soybeans were the worst at extracting all the components examined in this study. FADTS was the best at extracting lipid and Ca, intermediate at extracting protein, carbohydrate, oligosaccharides, Fe, phytic acid, and tannins, which were all enhanced by the prolonging of frozen days. Soymilk from 4 days FADTS contained the similar protein content and higher Fe content compared to the soymilk from soaked soybeans. In addition, soymilk from 4 days FADTS possessed the intermediate TIA and CIA and the highest in vitro protein digestibility compared to soymilks from untreated and soaked soybeans. Totally, it is considered that the 4 days FADTS, an instant soybean product, should be a good alternative for the soymilk making by soymilk grinding, which is not only good at the convenience of soymilk making, but also good at the nutritional properties of soymilk.

## CONFLICT OF INTEREST

No competing conflicts of interest existed among all authors.
